# Generation of Functional Cardiomyocytes from the Synoviocytes of Patients with Rheumatoid Arthritis via Induced Pluripotent Stem Cells

**DOI:** 10.1038/srep32669

**Published:** 2016-09-09

**Authors:** Jaecheol Lee, Seung Min Jung, Antje D. Ebert, Haodi Wu, Sebastian Diecke, Youngkyun Kim, Hyoju Yi, Sung-Hwan Park, Ji Hyeon Ju

**Affiliations:** 1 Division of Cardiology, Department of Medicine, Stanford University School of Medicine, Stanford, CA, USA; 2 Institute for Stem Cell Biology and Regenerative Medicine, Stanford University School of Medicine, Stanford, CA, USA; 3 Stanford Cardiovascular Institute, Stanford University School of Medicine, Stanford, CA, USA; 4 Division of Rheumatology, Department of Internal Medicine, Yonsei University College of Medicine, Seoul, Republic of Korea; 5 Department of Cardiology and Pneumonology, Göttingen University Medical Center, Göttingen, Germany; 6 DZHK (German Center for Cardiovascular Research), partner site Göttingen, Germany; 7 Max Delbrück Center, Berlin, Germany Berlin Institute of Health, Berlin, Germany; 8 Division of Rheumatology, Department of Internal Medicine, College of Medicine, Seoul St. Mary’s Hospital, The Catholic University of Korea, Seoul, South Korea

## Abstract

Cardiovascular disease is a leading cause of morbidity in rheumatoid arthritis (RA) patients. This study aimed to generate and characterise cardiomyocytes from induced pluripotent stem cells (iPSCs) of RA patients. Fibroblast-like synoviocytes (FLSs) from patients with RA and osteoarthritis (OA) were successfully reprogrammed into RA-iPSCs and OA-iPSCs, respectively. The pluripotency of iPSCs was confirmed by quantitative reverse transcription-polymerase chain reaction and immunofluorescence staining. Established iPSCs were differentiated into cardiomyocytes using a small molecule-based monolayer differentiation protocol. Within 12 days of cardiac differentiation from patient-specific and control-iPSCs, spontaneously beating cardiomyocytes (iPSC-CMs) were observed. All iPSC-CMs exhibited a reliable sarcomeric structure stained with antibodies against cardiac markers and similar expression profiles of cardiac-specific genes. Intracellular calcium signalling was recorded to compare calcium-handling properties among cardiomyocytes differentiated from the three groups of iPSCs. RA-iPSC-CMs had a lower amplitude and a shorter duration of calcium transients than the control groups. Peak tangential stress and the maximum contractile rate were also decreased in RA-iPSC-CMs, suggesting that contractility was reduced. This study demonstrates the successful generation of functional cardiomyocytes from pathogenic synovial cells in RA patients through iPSC reprogramming. Research using RA-iPSC-CMs might provide an opportunity to investigate the pathophysiology of cardiac involvement in RA.

Rheumatoid arthritis (RA) is a systemic autoimmune disease with unknown aetiology and is characterised by inflammatory polyarthritis^1^. Systemic inflammation often affects other organs, such as the kidney, lung and heart. Cardiovascular disease (CVD) is the most commonly encountered comorbidity and is the leading cause of mortality and morbidity in patients with RA^2^,^3^,^4^. The prevalence of ischemic heart disease and congestive heart failure is significantly higher in patients with RA than in age- and sex-matched controls^4^,^5^,^6^,^7^. Recent advances in imaging modalities, such as echocardiography and cardiac magnetic resonance, revealed that RA patients who are asymptomatic for CVD exhibit subclinical myocardial dysfunctions^8^,^9^,^10^. Although the increased risk of myocardial dysfunction in RA is associated with atherosclerosis accelerated by chronic inflammation, factors unique to RA would partly contribute to the development of heart failure.

To delineate the pathophysiology of RA in the myocardium, the ideal approach would be to perform disease modelling using a patient’s own cells or tissues. Although animal research has provided a better understanding of inflammatory arthritis in RA, animal models still have a limited value for studying the systemic involvement of RA^11^. Cardiomyocytes reflecting the myocardial pathology are directly attainable from a patient’s myocardium. However, tissue biopsy only secures a small number of target cells and involves the patient undergoing an invasive procedure. Ethical issues also hinder biopsies being taken from patients without overt myocarditis. Thus, acquisition of a patient’s own tissues is poorly applicable for research purposes.

Recently, disease modelling with patient-specific induced pluripotent stem cells (iPSCs) has been highlighted as a promising alternative to circumvent these limitations. iPSCs, pluripotent cells derived from adult somatic cells, were introduced by Yamanaka in 2006. Yamanaka and colleagues reprogrammed mature somatic cells into autologous stem cells using four canonical transcription factors, namely, OCT4, KLF4, SOX2 and c-MYC^12^. iPSCs have characteristics equivalent to embryonic stem cells (ESCs), which are able to proliferate indefinitely and differentiate into any target cells. The pathologic features of a certain disease would be recapitulated by pathogenic cells differentiated from patient-derive iPSCs. Disease modelling with iPSC technologies was primarily attempted for inherited diseases, such as familial dilated cardiomyopathy, catecholaminergic polymorphic ventricular tachycardia and spinal muscular atrophy^13^,^14^,^15^.

Although the simulation of chronic diseases with patient-specific iPSCs has not been validated, it would have a potential advantage in systemic diseases with a genetic predisposition. Previously, the development and progression of RA showed an association with histocompatibility leukocyte antigens, so-called shared epitopes^16^. Earlier studies also support the role of genetic background in the systemic involvement of RA^17^,^18^,^19^. Thus, we tried to extend RA synoviocyte-derived iPSCs beyond the joints. Here, we report the effective differentiation of cardiac cells from RA patient-specific iPSCs and describe their characterisation as functional cardiomyocytes.

## Results

### Generation and characterisation of iPSCs

**Figure 1:**
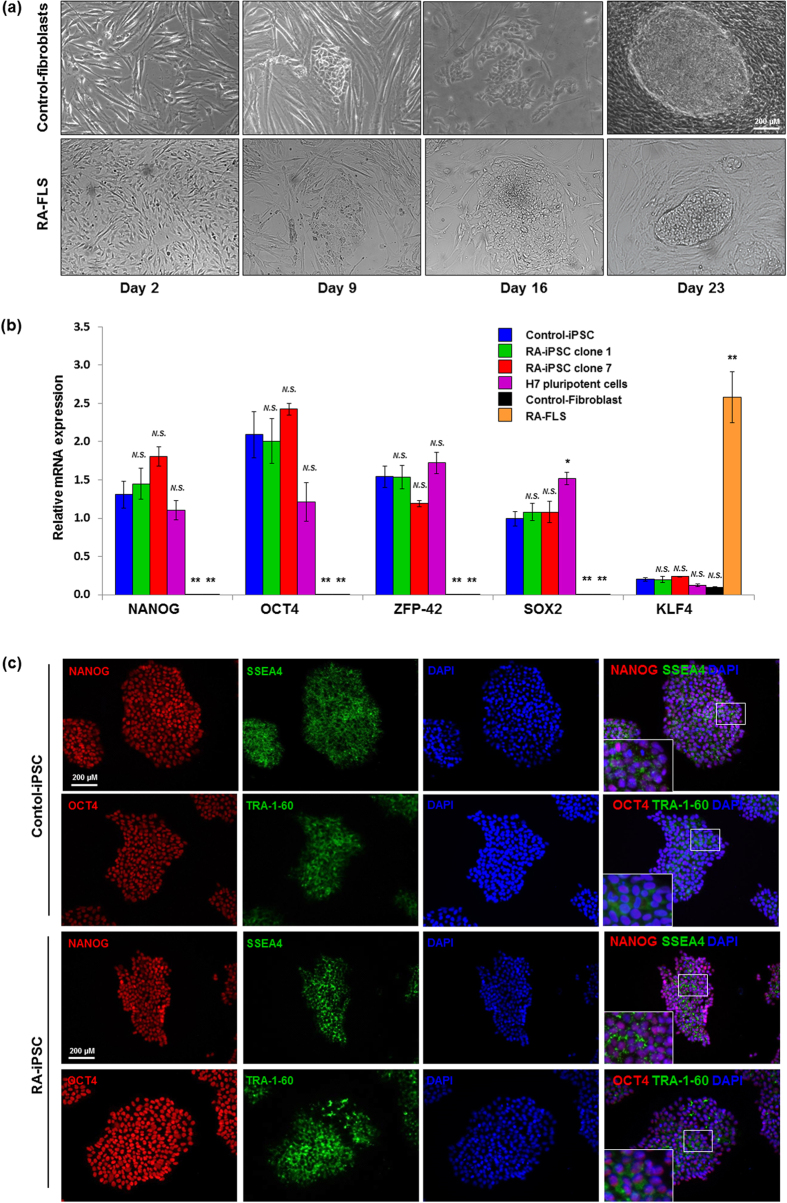
Generation and characterisation of RA patient-specific iPSCs. (**a**) RA-FLSs and healthy dermal fibroblasts were reprogrammed into iPSCs via lentiviral transduction of *oct4*, *sox2*, *nanog* and *c-myc* (scale bar, 200 μm). (**b**) The mRNA levels of pluripotency genes in primary somatic cells, iPSCs and H7 cells were measured by quantitative reverse transcription-polymerase chain reaction. Data represent mean values of three independent experiments ± SEM. NS, not significant; *P < 0.05; **P < 0.01. (**c**) The expression of pluripotency markers in iPSC clones was confirmed by immunofluorescence staining with antibodies against NANOG, SSEA, OCT4 and TRA-1-60. The right-hand panels show merged images of iPSC colonies stained with specific antibodies (red and green) and DAPI (blue). RA-FLSs, fibroblast-like synoviocytes obtained from patients with rheumatoid arthritis (RA); RA-iPSCs, induced pluripotent stem cells (iPSCs) derived from RA patients; control-iPSCs, iPSCs derived from a healthy control subject.

Fibroblast-like synoviocytes (FLSs) of patients with RA (n = 3) and osteoarthritis (OA) (n = 3) and dermal fibroblasts of healthy subjects (n = 3) were efficiently reprogrammed by lentiviral transduction of *oct4*, *sox2*, *nanog* and *c-myc* (Fig. 1a). On day 16, stem cell-like colonies appeared in the culture dishes of primary cells and were transferred onto a feeder-free growth layer. Subsequently, iPSC clones were established from patients with RA (RA-iPSCs) and patients with OA (OA-iPSCs), as well as a healthy control subject (control-iPSCs). All iPSCs had a normal chromosome pattern of 44 + XX or 44 + XY (Supplementary Figure 1).

Established iPSCs were analysed by quantitative reverse transcription-polymerase chain reaction (qRT-PCR) to determine their expression of *NANOG*, *OCT4*, *ZFP-42* and *SOX2*. All iPSC lines exhibited similar expression levels of the pluripotency markers (Fig. 1b), irrespective of the primary cells. The pluripotency of the iPSC clones was further characterised by immunofluorescence staining. RA-iPSCs, OA-iPSCs and control-iPSCs were positively stained for well-established pluripotency markers, including OCT4, NANOG, SSEA4 and TRA-1-60 (Fig. 1c). No significant differences were discerned among iPSC lines in terms of their expression of pluripotency markers.

### Differentiation of iPSCs into cardiomyocytes

**Figure 2:**
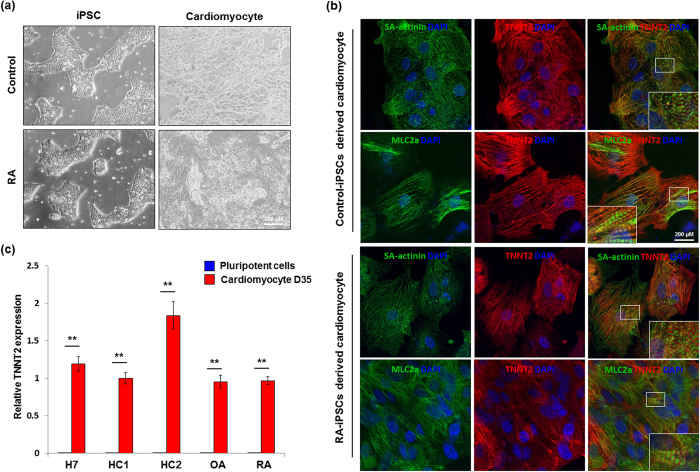
Differentiation of patient-specific iPSCs into cardiomyocytes. (**a**) Beating cardiomyocytes were generated from control-iPSCs and RA-iPSCs after 12 days of cardiac differentiation. (**b**) The cellular structure and expression of cardiac markers in iPSC-derived cardiomyocytes were confirmed by immunofluorescence staining and confocal microscopy. Control-iPSC-CMs and RA-iPSC-CMs both showed characteristic sarcomeric structures and positive staining with antibodies against SA-actinin, TNNT2 and MLC2a (scale bar, 200 μm). (**c**) The mRNA level of a cardiac-specific gene, TNNT2, before and after cardiac differentiation in various cells was determined by quantitative reverse transcription-polymerase chain reaction. Data represent mean values determined in three independent experiments ± SEM. **P < 0.01. RA, rheumatoid arthritis; iPSCs, induced pluripotent stem cells; HC, healthy control; OA, osteoarthritis; SA-actinin, sarcomeric alpha-actinin; MLC2a, myosin light chain 2a; TNNT2, cardiac troponin T type 2.

All iPSC clones were cultured for 20 passages to render the cells epigenetically closer to ESCs^20^,^21^. Established iPSCs and H7 cells were successfully differentiated into cardiomyocytes, with beating cells observed on day 12 of cardiac differentiation (Fig. 2a and Supplementary Movie 1). We confirmed the purity of cardiomyocytes derived from iPSCs (iPSC-CMs) by performing flow cytometry with a cardiac-specific marker, cardiac troponin T type 2 (TNNT2). Using glucose deprivation to enhance purification, we could generate TNNT2-positive cardiomyocytes with 80–87% purity (Supplementary Figure 2). To further characterise the quality of iPSC-CMs, we measured the gene expression levels of TNNT2 and myosin heavy chain, α-isoform (MYH6), by qRT-PCR (Fig. 2b and Supplementary Figure 3). The analysis revealed no significant difference in mRNA expression among cardiomyocytes derived from RA-iPSCs, OA-iPSCs, control-iPSCs and H7 cells (designated RA-iPSC-CMs, OA-iPSC-CMs, control-iPSC-CMs and H7-iPSC-CMs, respectively). On the other hand, these genes were scarcely detectable in parental iPSCs by qRT-PCR analysis.

The sarcomeric structures were similar in RA-iPSC-CMs and control-iPSC-CMs. Furthermore, immunofluorescence staining of RA-iPSC-CMs and control-iPSC-CMs showed comparable expression patterns of functional cardiac markers such as TNNT2, myosin light chain 2a (MLC2a) and sarcomeric alpha-actinin (SA-actinin) (Fig. 2c).

### Characterisation of patient-specific iPSC-derived cardiomyocytes

**Figure 3:**
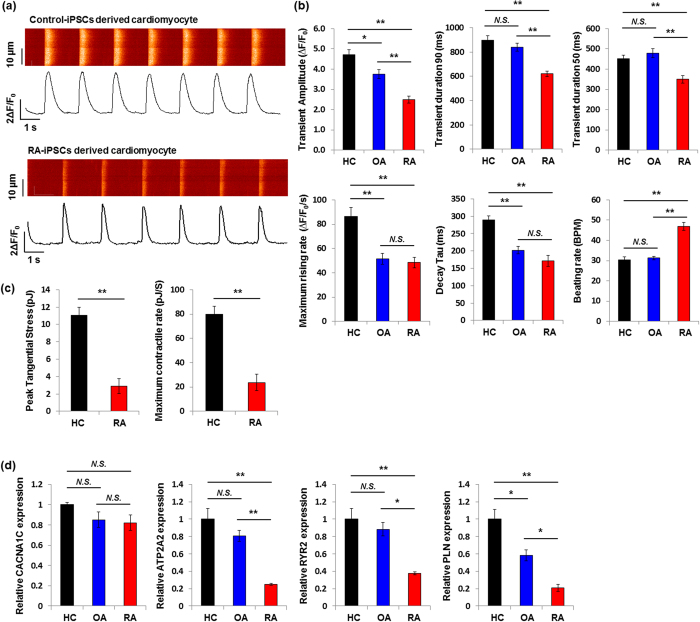
Characterisation of cardiomyocytes derived from patient-specific iPSCs. (**a**) Representative line-scan images and spontaneous calcium signalling of RA-iPSC-CMs and control-iPSC-CMs showed regular calcium transients. (**b**) The calcium-handling properties of RA-iPSC-CMs, OA-iPSC-CMs and control-iPSC-CMs were compared by examining calcium-handling parameters. (**c**) Contractility measurements were recorded on single, spontaneously beating cardiomyocytes derived from RA-iPSCs and control-iPSCs. (**d**) Expression of calcium signalling-related genes was measured in iPSC-CMs using quantitative reverse transcription-polymerase chain reaction. Data represent mean values determined in three independent experiments ± SEM. NS, not significant; *P < 0.05; **P < 0.01. RA, rheumatoid arthritis; RA-iPSCs, induced pluripotent stem cells (iPSCs) derived from RA patients; HC, healthy control; control-iPSCs, iPSCs derived from a healthy control subject; CACNA1C, voltage-dependent L type calcium channel subunit alpha 1C; ATP2A2, cardiac muscle calcium-transporting ATPase; RYR2, cardiac ryanodine receptor 2; PLN, phospholamban.

Because calcium is a crucial messenger in myocardial function, analysis of calcium handling allows insights into electrical excitation and mechanical contraction of cardiomyocytes^22^. Therefore, we next compared the calcium-handling parameters of RA-iPSC-CMs and other control cells. Representative traces showed a regular pattern of calcium transients in RA-iPSC-CMs and control-iPSC-CMs (Fig. 3a), with a faster beating rate of ~45 beats/min in RA-iPSC-CMs (Fig. 3b). The calcium-handling properties of RA-iPSC-CMs showed distinct features compared with those of control-iPSC-CMs and OA-iPSC-CMs. The maximum rising rate of the calcium transient and the decay Tau were comparable between RA-iPSC-CMs and OA-iPSC-CMs, whereas the parameters were significantly elongated in control-iPSC-CMs. RA-iPSC-CMs had a significantly reduced amplitude and a shorter duration of calcium transients than the other cells (Fig. 3b). The peak tangential stress and maximum contractile rate were also decreased in RA-iPSC-CMs (Fig. 3c). In line with these findings, we determined the expression of calcium signalling-related genes in iPSC-CMs. The expression level of voltage-dependent L type calcium channel subunit alpha 1C was not altered among the three groups (Fig. 3d). However, several genes critically involved in cardiomyocyte-specific calcium handling, such as cardiac muscle calcium-transporting ATPase (ATP2A2), cardiac ryanodine receptor 2 (RYR2) and phospholamban (PLN), were significantly down-regulated in RA-iPSC-CMs compared with the other groups (Fig. 3d).

Because FLSs are a major pathogenic component of RA, we hypothesised that the expression of inflammatory genes might differ between cardiomyocytes derived from RA-FLSs and cardiomyocytes derived from OA-FLSs and healthy dermal fibroblasts. Thus, we determined the expression of interleukin (IL)-6, IL-1, tumour necrosis factor (TNF)-α and TNF receptor 2 (TNFR2) to assess whether the inflammatory signature of RA-FLSs was retained after their differentiation into cardiomyocytes via iPSCs. Although RA-FLSs showed significantly higher expression levels of IL-6, IL-1, TNF-α and TNFR2 than the control groups, this difference was not observed following their differentiation into iPSCs and iPSC-CMs, suggesting that the inflammatory profiles are abolished during iPSC reprogramming and differentiation (Supplementary Figure 4).

## Discussion

In the present study, we successfully reprogrammed pathogenic FLSs from RA patients into iPSCs and subsequently differentiated these cells into functional cardiomyocytes. To our knowledge, this is the first attempt to generate functional cardiomyocytes from RA patient-specific iPSCs. RA-iPSC-CMs demonstrated a characteristic sarcomeric structure and expressed various cardiac-specific markers, comparable to control-iPSC-CMs and OA-iPSC-CMs. However, calcium-handling properties differed between RA-iPSC-CMs and the other groups.

Regardless of the parental cells, all iPSC lines showed similarities in terms of their reprogramming efficiency and resultant pluripotency. The reprogramming process can be affected by several factors, such as reprogramming methods and primary somatic cells^23^. The characteristics of the original cells can enhance or disturb reprogramming into iPSCs^24^,^25^. Synovial cells from RA patients are expected to undergo more epigenetic changes than other somatic cells. However, reprogramming into iPSCs was comparable between healthy dermal fibroblasts and pathogenic FLSs. Moreover, expression of inflammatory proteins in RA synoviocytes, such as IL-6, IL-1 and TNF-α, was lost after reprogramming into iPSCs.

Cardiomyocytes differentiated from control-iPSCs and patient-derived iPSCs displayed a sarcomeric structure with spontaneous contractile activity. We confirmed the cardiac features of iPSC-CMs by investigating expression of the sarcomeric components TNNT2 and MYH6. A substantial number of differentiated cells was positive for the cardiac marker TNNT2 in both groups, suggesting that cardiac differentiation from iPSCs was successful. The proportion of TNNT2-positive cardiomyocytes differentiated from iPSCs was comparable with previous reports.

Although the reprogramming and differentiation processes showed no significant differences between the groups, RA-iPSC-CMs had different calcium-handling properties compared with OA-iPSC-CMs and control-iPSC-CMs. The most prominent finding was the low amplitude and short duration of calcium transients in RA-iPSC-CMs, suggesting that calcium currents were decreased. The contractile activity represented by peak tangential stress and the maximum contractile rate was also decreased in RA-iPSC-CMs. Reduced expression of ATP2A2 and RYR2, which encode the calcium channels in cardiac myocytes, may account for the calcium-handling defect in RA-iPSC-CMs. However, the effect of down-regulated PLN can be confusing. PLN is a membrane protein that inhibits sarcoplasmic/endoplasmic reticulum calcium-ATPase, which pumps cytoplasmic calcium into the sarcoplasmic reticulum. Inactivation of PLN in RA-iPSC-CMs enhances the storage of calcium in the sarcoplasmic reticulum, leading to promotion of relaxation and the following contraction. Thus, the combined effect of reduced expression of calcium handling-related genes in RA-iPSC-CMs requires further research.

Experimental studies of cardiomyopathies have been restricted due to the limitations of cellular resources; therefore, the electrophysiological properties of the myocardium in RA patients have not been elucidated. We only infer cardiogenic dysfunction based on the electrocardiographic abnormalities. A population-based study revealed prolongation of the corrected QT interval in RA patients^26^. Based on the association between the C-reactive protein level and QT interval^27^,^28^, the inflammatory burden is considered to prolong the QT interval. However, the underlying mechanism and the myocardial pathology that is susceptible to inflammation remain to be determined. iPSC technologies would allow evaluation of myocardial dysfunction in RA, as well as the pathologic response of the myocardium in the inflammatory milieu in RA.

In this study, we employed OA-iPSCs as another comparator. Patients with OA have been frequently regarded as controls for RA patients^29^. RA and OA often share similarities in terms of joint involvement; however, the principal pathophysiology of arthritis differs^30^. RA is an inflammatory disease characterised by pannus formation, while OA is a degenerative disease affected by mechanical injury and pressure overload. This disparity makes it possible to investigate the effect of the inflammatory burden on the pathophysiology of RA.

The limitation of this study is that a relatively small sample size was used to reveal the pathophysiologic features of RA-related cardiomyopathies. Three patients might be insufficient to show the disease characteristics, rather than individual characteristics. However, calcium-handling properties consistently differed between RA-iPSC-CMs and the other groups, suggesting that they reflect the disease. The recruitment of more patients would improve the power to simulate heart disease in RA. In addition, the effect of chronic inflammation was not fully considered in this model. Although disease modelling using iPSC technologies has mainly focused on primary myocardial disease^31^, simulation of cardiomyopathies secondary to metabolic disease can be attempted. Cardiomyocytes differentiated from patient-derived iPSCs can be manipulated by environmental stress to recapitulate the disease phenotype^32^. The response of RA-iPSC-CMs to inflammatory molecules might provide a better understanding of the cardiac involvement of RA.

Nevertheless, to our knowledge, this is the first study to report the successful differentiation of iPSCs derived from RA patients into cardiomyocytes. We confirmed the general features of the differentiated cardiomyocytes by several reliable methods including the following: (1) visualisation of beating cells, (2) analysis of the expression of cardiac-specific markers via qRT-PCR and immunostaining, and (3) calcium imaging to show relevant calcium transient waveforms. These iPSC-CMs would provide more opportunities for disease modelling and drug screening.

In conclusion, we generated functional cardiomyocytes using iPSCs derived from synoviocytes of patients with RA and OA. iPSC-CMs demonstrate a possible application of patient-derived iPSCs in rheumatology. This concept can be theoretically extended to other cell types, including osteocytes, chondrocytes, immune cells and neurons. We anticipate that ongoing progress in iPSC research and technologies will provide valuable information in real clinical settings of rheumatology.

## Materials and Methods

### Derivation of human iPSCs

FLSs were isolated from the synovial tissues of three patients with RA and three patients with OA and used to generate patient-specific iPSCs. The synovial tissues were obtained during a surgical procedure for therapeutic purposes and stored anonymously in the tissue bank of the Catholic Rheumatism Research Center prior to reprogramming. Tissues were minced and resuspended in Dulbecco’s modified Eagle’s medium (DMEM; Gibco, Invitrogen Corporation) containing 0.01% collagenase, and then incubated for 4 h at 37 °C with vigorous shaking. Cells were washed and resuspended in DMEM supplemented with 20% foetal bovine serum (Gibco) and 1% penicillin/streptomycin solution (Gibco). Cells were cultured until the adherent fibroblasts became confluent. Dermal fibroblasts were acquired from a healthy volunteer with informed consent and reprogrammed into control-iPSCs for comparative analyses.

Reprogramming of somatic cells into iPSCs was performed as previously described^33^. Briefly, the primary cells were incubated with a lentiviral construct encoding *oct4*, *sox2*, *nanog* and *c-myc* in the presence of polybrene. The medium was replaced after 24 h, and then every other day for the next 6 days. On day 7, cells were transferred onto Matrigel-coated 6-well plates containing DMEM/GlutaMAX™ (Life Technologies) supplemented with 10% foetal bovine serum and a Rho kinase inhibitor, Y-27632 (10 mM prepared in 100 μl of Dulbecco’s phosphate-buffered saline) (Selleck Chemicals). After the cells adhered, the medium was changed to chemically defined Essential 8™ medium (Life Technologies). On day 16, colonies were manually picked and transferred to a fresh Matrigel-coated culture dish. The medium was changed daily, and cells were passaged every 3–4 days using Accutase Cell Detachment Solution (Global Cell Solutions).

#### Ethics

All procedures and methods involving human samples were in accordance with approved guidelines. We received informed consents from all of the participants. This study was approved by the Institutional Review Board of the Catholic University of Korea (KC13TISI0577).

### Differentiation of iPSCs into cardiomyocytes

We utilised a chemically defined monolayer differentiation protocol for cardiac differentiation^34^,^35^. Briefly, iPSCs at ~90% confluency were incubated with differentiation basal medium, namely, RPMI 1640 medium (Invitrogen) containing 2% B27 supplement minus insulin (Invitrogen). CHIR99021, a selective glycogen synthase kinase 3β inhibitor (6-[[2-[[4-(2,4-dichlorophenyl)-5-(5-methyl-1*H*-imidazol-2-yl)-2-pyrimidinyl]amino]ethyl]amino]-3-pyridinecarbonitrile; 10 mM in 15 μl of dimethyl sulfoxide), was also added to the differentiation basal medium. On day 2, the medium was replaced with differentiation basal medium lacking CHIR99021, and, on day 3, the Wnt antagonist IWR-1 (4-(1,3,3a,4,7,7a-hexahydro-1,3-dioxo-4,7-methano-2H-isoindol-2-yl)-N-8-quinolinyl-benzamide; 10 mM in 6 μl of dimethyl sulfoxide) was added to the medium. After another 48 h, the medium was replaced with differentiation basal medium without any inhibitors. On day 7, the cells were incubated with complete cardiomyocyte medium, namely, RPMI 1640 medium containing 2% B27 supplement plus insulin (Invitrogen). The medium was changed every other day. Monolayers of iPSC-CMs were cultured for ~30 days and subsequently dissociated for experimental use with TrypLE™ Express (Life Technologies).

### Immunofluorescence staining and confocal microscopy

Established iPSCs were fixed with 4% paraformaldehyde prepared in phosphate-buffered saline. Following permeabilisation with 0.3% Triton X-100, iPSCs were stained with primary antibodies against OCT3/4, SSEA4, NANOG (all from Santa Cruz Biotechnology, Inc.) and TRA-1-60 (Chemicon). iPSC-CMs were then passaged onto Matrigel-coated 12 mm glass coverslips, followed by staining with antibodies against TNNT2 (Thermo Scientific and Abcam), MLC2a (Santa Cruz Biotechnology) and SA-actinin (Sigma Chemical Co). After reaction with the primary antibodies, cells were incubated with the appropriate Alexa Fluor-conjugated secondary antibodies (Santa Cruz Biotechnology or Life Technologies).

Images of the stained cells were obtained under a bright field microscope (Leica). Confocal images were taken using a 63× Plan-Apochromat oil immersion objective (Carl Zeiss) and a LSM 510 Meta confocal microscope (Carl Zeiss). Images were analysed using ZEN software (Carl Zeiss) and ImageJ software (National Institutes of Health).

### RNA extraction and qRT-PCR

Total RNA was isolated using the RNeasy Plus Mini Kit (Qiagen). The reverse transcription reaction was performed using the iScript™ cDNA Synthesis Kit (Bio-Rad), and qRT-PCR was performed using iQ^®^ SYBR™ Green Supermix (Bio-Rad) and a CFX96™ real-time PCR detector (Bio-Rad). Relative mRNA levels were normalised against those of 18S mRNA for each reaction. The primers for qRT-PCR are presented in the Supplementary Table.

### Intracellular calcium imaging

iPSC-CMs were plated onto Matrigel-coated 8-well Lab-Tek^®^ II coverglass imaging chambers (Thermo Fisher Scientific, Inc.) at a density of 20,000 cells per well. After 3–4 days, cells were loaded with 5 μM Fluo-4 AM prepared in Tyrode’s solution (140 mM NaCl, 5.4 mM KCl, 1 mM MgCl_2_, 10 mM glucose, 1.8 mM CaCl_2_ and 10 mM HEPES, pH adjusted to 7.4 with NaOH at room temperature) for 5 min at 37 °C. Next, cells were washed three times with pre-warmed Tyrode’s solution. Spontaneous calcium signalling in iPSC-CMs was analysed using a LSM 510 Meta confocal microscope equipped with a 63× Plan-Apochromat oil immersion objective. Signalling was recorded in line-scanning mode (512 pixels × 1920 lines) at a speed of 3.2 μs/pixel. A custom-made script based on the Interactive Digital Language was used for image processing. The extracellular background was subtracted from the calcium signalling, and the calcium signal was normalised to the intracellular basal line. The transient amplitude was expressed as ∆F/F_0_.

### Statistical analysis

All quantifiable experimental data are presented as mean ± standard error of mean (SEM). Statistical significance was determined by the Mann-Whitney U test or an analysis of variance followed by Bonferroni’s post hoc test. *P*-values <0.05 were considered statistically significant. Data analysis was performed using SAS 9.1 software (SAS Institute).

## Additional Information

**How to cite this article**: Lee, J. *et al*. Generation of Functional Cardiomyocytes from the Synoviocytes of Patients with Rheumatoid Arthritis via Induced Pluripotent Stem Cells. *Sci. Rep*. **6**, 32669; doi: 10.1038/srep32669 (2016).

## Supplementary Material

Supplementary Information

Supplementary Video S1
