# Supplementary material for: Generation of Functional Cardiomyocytes from the Synoviocytes of Patients with Rheumatoid Arthritis via Induced Pluripotent Stem Cells
Source: Sci Rep. 2016 Sep 9;6:32669. doi: 10.1038/srep32669 (PMC5016736; doi:10.1038/srep32669)
Supplement: Supplementary Information [file srep32669-s1.pdf]

# Supplementary Information

## Generation of Functional Cardiomyocytes from the Synoviocytes of Patients with Rheumatoid Arthritis via Induced Pluripotent Stem Cells

Jaecheol Lee[1,2,3†], Seung Min Jung[4†], Antje D. Ebert[1,2,3†], Haodi Wu[1,2,3], Sebastian Diecke[1,2,3], Youngkyun Kim[5], Hyoju Yi[5], Sung-Hwan Park[5] and Ji Hyeon Ju[5*]

[†]These authors contributed equally to this study and should be considered joint first authors.

[1]Division of Cardiology, Department of Medicine, Stanford University School of Medicine, Stanford, CA, USA

[2]Institute for Stem Cell Biology and Regenerative Medicine, Stanford University School of Medicine, Stanford, CA, USA

[3]Stanford Cardiovascular Institute, Stanford University School of Medicine, Stanford, CA, USA

[4]Division of Rheumatology, Department of Internal Medicine, Yonsei University College of Medicine, Seoul, Republic of Korea

[5]Division of Rheumatology, Department of Internal Medicine, College of Medicine, Seoul St. Mary's Hospital, The Catholic University of Korea, Seoul, South Korea

***Corresponding author:**

Ji Hyeon Ju, MD PhD

CiSTEM laboratory, Convergent Research Consortium for Immunologic Disease, Division of Rheumatology, Department of Internal Medicine, College of Medicine, Seoul St. Mary's Hospital, The Catholic University of Korea, 222 Banpo-daero, Seocho-gu, Seoul 06591, Republic of Korea

Tel: 82-2-2258-6893, Fax: 82-2-3476-2274, E-mail address: juji@catholic.ac.kr

RA iPSC 1

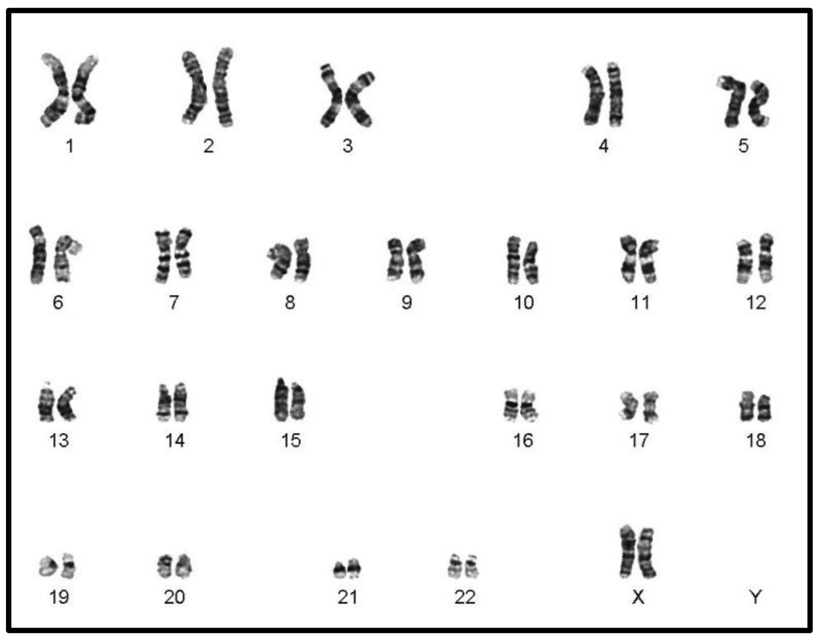


RA iPSC 2

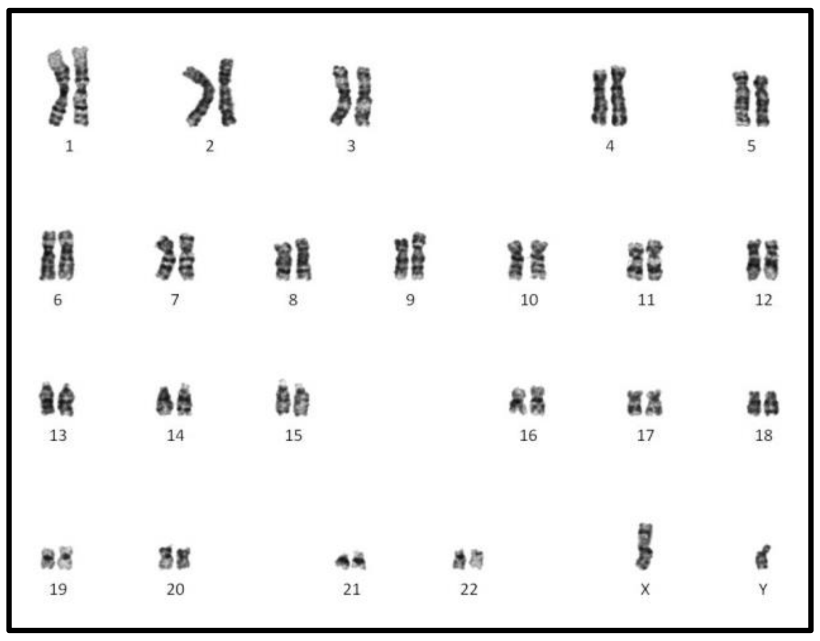


OA iPSC 1

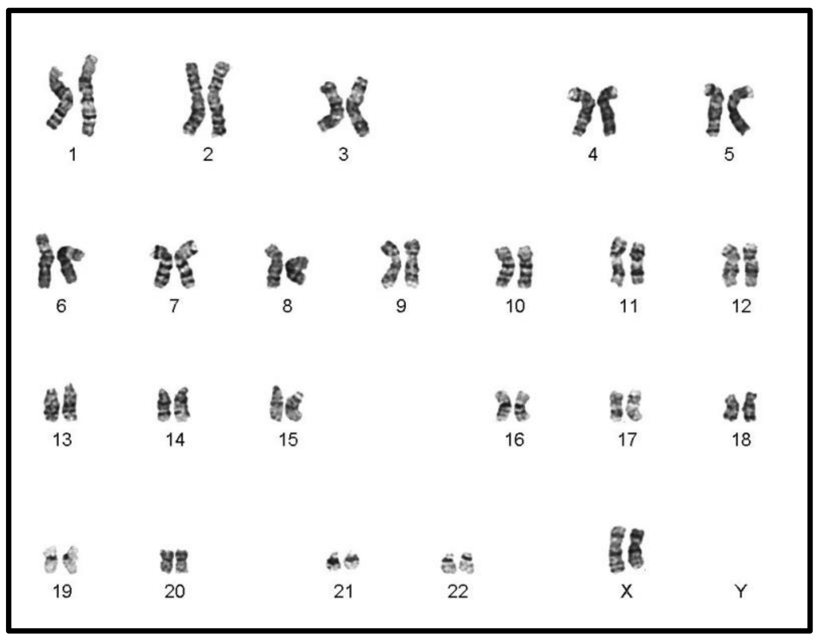


OA iPSC 2

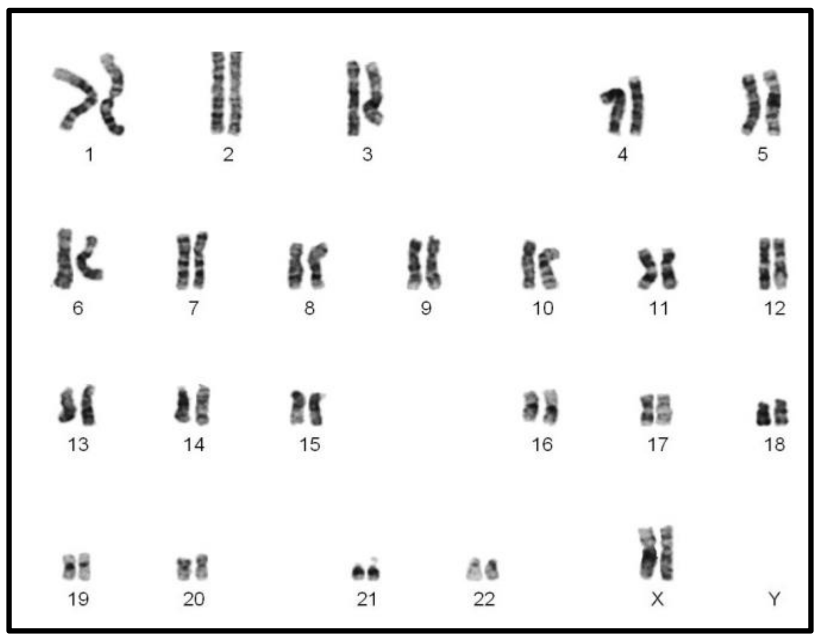


Lee *et al*. **Supplementary figure S1**

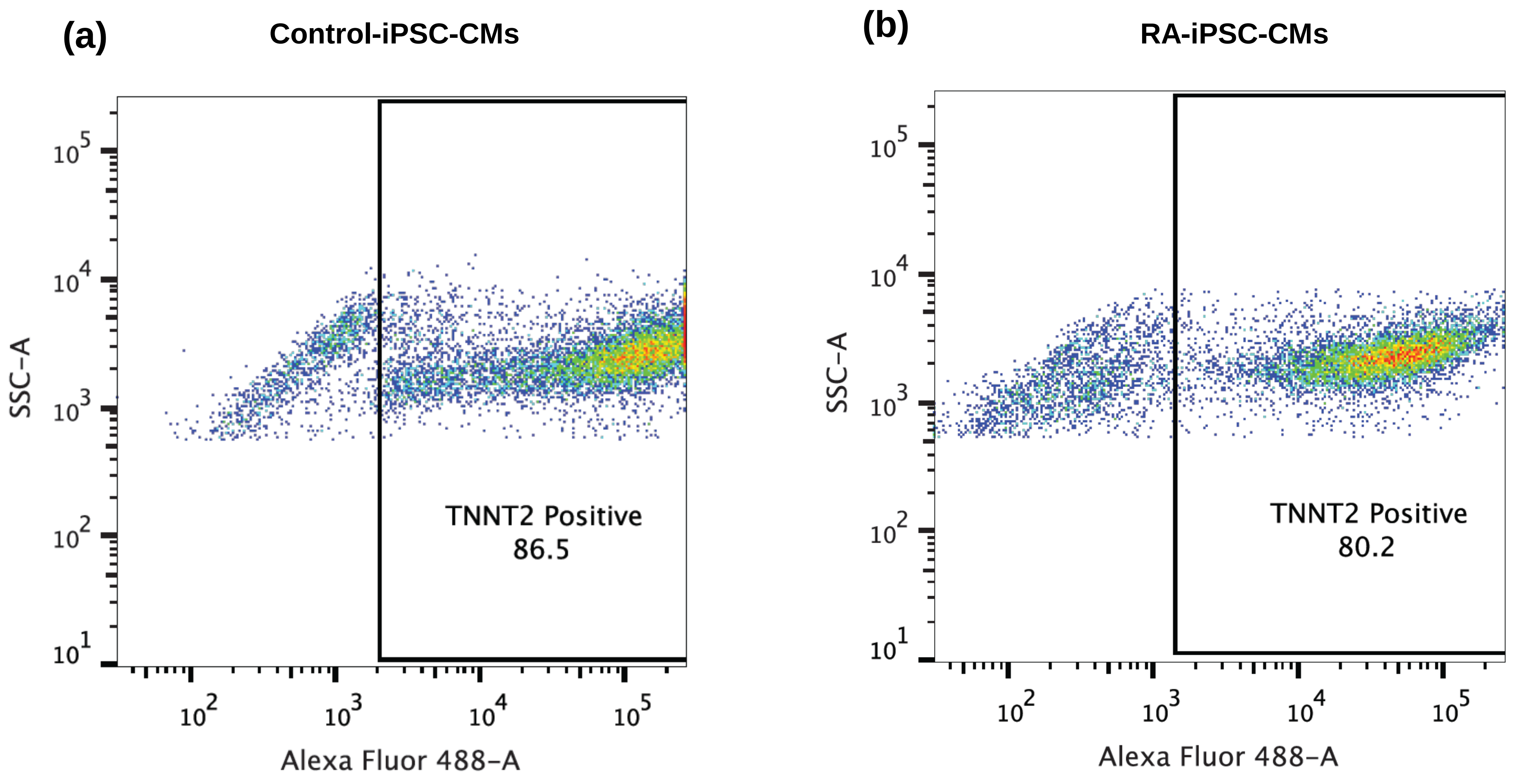


Lee *et al*. **Supplementary figure S2**

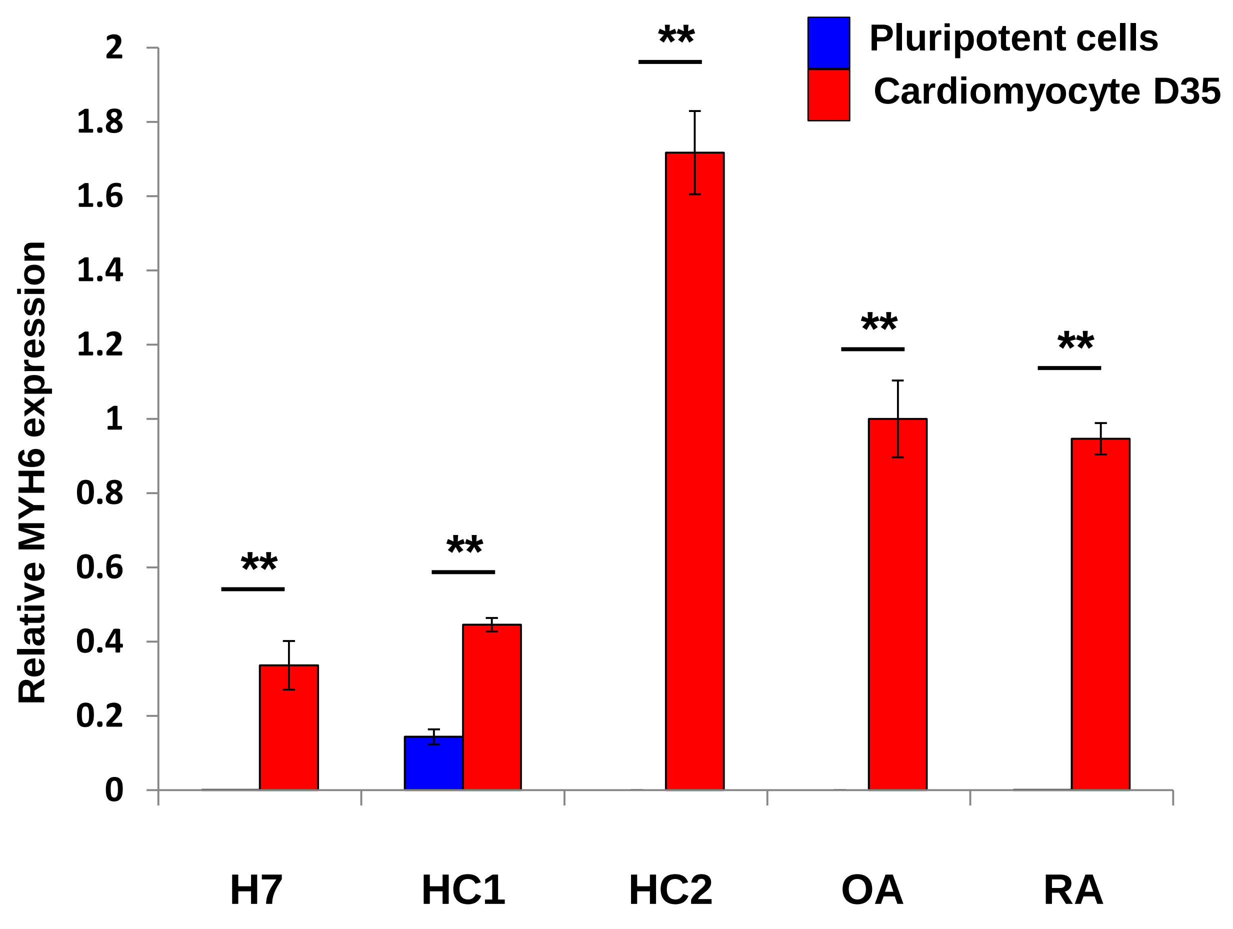


Lee *et al*. **Supplementary figure S3.**

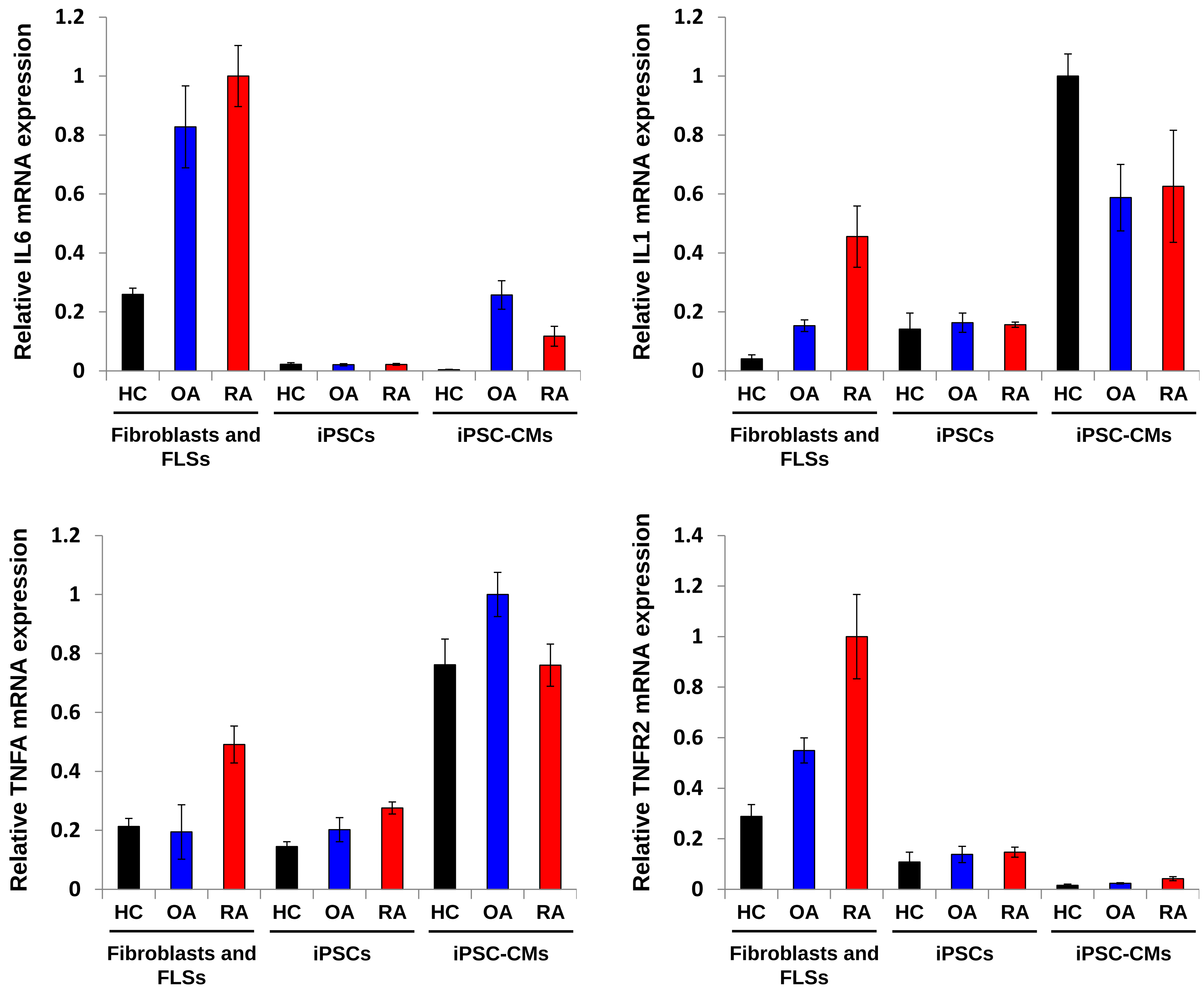


Lee *et al*. **Supplementary figure S4**

| Gene name | NCBI number | Direction | Sequences |
|---|---|---|---|
| POU5F1 | NM_002701 | Forward | CCATGCATTCAAACTGAGGTG |
| | | Reverse | CCTTTGTGTTCCCAATTCCTTC |
| NANOG | NM_024865.2 | Forward | TTCACTGTGTTAGCCAGGATG |
| | | Reverse | ATGTCTTTTCTAGGCAGGGC |
| SOX2 | NM_003106 | Forward | GAGAAGTTTGAGCCCCAGG |
| | | Reverse | AGAGGCAAACTGGAATCAGG |
| ZFP42 | NM_174900.3 | Forward | TTCTCCTTTGTTTTACGTTTGGG |
| | | Reverse | AGATCACACCATTGCACTCC |
| KFL4 | NM_004235.4 | Forward | ACCTACACAAAGAGTTCCCATC |
| | | Reverse | TGTGTTTACGGTAGTGCCTG |
| IL1 alpha | NM_000575.3 | Forward | CAGGAGCTGCCAAGTATTC |
| | | Reverse | TCTAGGAGGAAGGGAGAAATC |
| IL6 | NM_000600.3 | Forward | CCCAGGAGAAGATTCCAAAG |
| | | Reverse | GCTGCTTTCACACATGTTAC |
| TNF alpha | NM_000594.3 | Forward | CTGGTATGAGCCCATCTATCT |
| | | Reverse | GGGCAATGATCCCAAAGTAG |
| TNFRSF1B | NM_001066.2 | Forward | CAATGGGAGACACAGATTCC |
| | | Reverse | CTGGTTAACTGGGCTTCATC |

Lee *et al*. **Supplementary Table S1**

**Supplemental Information**

**Supplementary Figure 1.** Karyotypes of patient-specific induced pluripotent stem cells (iPSCs). Representative karyotypes of iPSCs derived from patients with rheumatoid arthritis (RA) and osteoarthritis (OA) were visualised by Giemsa staining.

**Supplementary Figure 2.** Purity of induced pluripotent stem cell (iPSC)-derived cardiomyocytes. Flow cytometry showed that 87% and 80% of cardiomyocytes derived from control-iPSCs and rheumatoid arthritis (RA)-iPSCs were positive for the cardiac-specific marker cardiac troponin T type 2 (TNNT2), respectively.

Control-iPSC-CMs, cardiomyocytes derived from control-iPSCs; RA-iPSC-CMs, cardiomyocytes derived from RA-iPSCs.

**Supplementary Figure 3.** Relative expression of myosin heavy chain in cardiomyocytes derived from induced pluripotent stem cells (iPSCs) and H7 cells. Expression of a cardiac-specific gene, myosin heavy chain, α-isoform (MYH6), was evaluated in various cells by quantitative reverse transcription-polymerase chain reaction. Data represent mean values determined in three independent experiments ± SEM. NS, not significant; $^{**}P < 0.01$.

HC, healthy control; OA, osteoarthritis; RA, rheumatoid arthritis.

**Supplementary Figure 4.** Inflammatory signatures of primary cells, iPSCs and iPSC-CMs. Expression profiles of inflammatory genes were determined in primary cells, iPSCs and cardiomyocytes derived from iPSCs by quantitative reverse transcription-polymerase chain

reaction. The inflammatory signatures of FLSs from RA patients were eliminated after reprogramming into iPSCs.

HC, healthy control; OA, osteoarthritis; RA, rheumatoid arthritis; iPSCs, induced pluripotent stem cells; FLSs, fibroblast-like synoviocytes; IL-1, interleukin-1; IL-6, interleukin-6; TNF-α, tumour necrosis factor-alpha; TNFR2, TNF receptor 2.

**Supplementary Table.** Primers of endogenous genes used for qRT-PCR. The sequences presented in the table were used to amplify endogenous genes in iPSCs. The primers targeted the 3’ or 5’ UTR of the respective mRNAs.

**Video legends**

**Supplementary movie 1**

Established induced pluripotent stem cells were successfully differentiated into cardiomyocytes. Beating cells began to be observed on day 12 of cardiomyocyte differentiation.
